# A Cross-Sectional Study of Electrophysiological Changes Occurring in Type II Diabetes Mellitus

**DOI:** 10.7759/cureus.28994

**Published:** 2022-09-09

**Authors:** Parikshit A Muley, Pranjali P Muley, Abhishek D Sambre, Ranjit S Ambad

**Affiliations:** 1 Physiology, Jawaharlal Nehru Medical College, Datta Meghe Institute of Medical Sciences, Wardha, IND; 2 Physiology, Datta Meghe Medical College and Shalinitai Meghe Hospital & Research Center, Datta Meghe Institute of Medical Sciences, Nagpur, IND; 3 Neurophysiology, Galaxy Vidarbha Diagnostics, Nagpur, IND; 4 Biochemistry, Datta Meghe Medical College and Shalinitai Meghe Hospital & Research Center, Datta Meghe Institute of Medical Sciences, Nagpur, IND

**Keywords:** neuropathy, peripheral neuropathy, nerve conduction study, glycaemic control, diabetes mellitus

## Abstract

Background

Diabetes is a long-term metabolic condition that results in high blood sugar levels from either reduced insulin production or diminished tissue sensitivity to insulin. Peripheral neuropathy is the most frequent consequence of diabetes. In this research project, with the aid of neurophysiological measures, we conducted a cross-sectional study to examine the impact of glycemic management on the physiological functioning of nerves, regardless of the duration of diabetes.

Objectives

The main objective of the study was to investigate the association between the degree of glycemic control and the severity of neurological changes. The study also aimed to clarify whether glycemic management, independent of the duration of diabetes, acts as an independent risk factor for the emergence of diabetic neuropathy.

Methodology

A total of 150 type 2 diabetic patients visiting the diabetic outpatient department were included in the study. The patients were divided into two groups: group A consisted of 90 subjects with HbA1c levels <10 and group B comprised 60 subjects with HbA1c levels >10. In the neurophysiology lab, an electrodiagnostic exam was conducted on the sensory (sural nerve) and motor (tibial nerve) parameters. Data on the neurophysiological parameters of the two groups were analyzed and compared.

Results

When the neurophysiological parameters of the two groups (group A having HbA1c <10 and group B having HbA1c >10) were analyzed, it was observed that group B had lower conduction velocity (CV) and amplitude potential than group A, with a significant statistical difference (p<0.05). It was also observed that sensory parameters were more affected than motor parameters.

Conclusion

Based on our findings, glycemic control is related to the severity of neuropathic changes.

## Introduction

Reduced insulin secretion or diminished tissue insulin sensitivity are the hallmarks of diabetes mellitus (DM), a metabolic illness that affects the metabolism of carbohydrates, lipids, and proteins [1]. Diabetes is a long-term metabolic disorder marked by an increase in blood glucose levels [2]. Hyperglycemia can develop in patients with diabetes when insulin is not produced at all or produced in insufficient quantities, or when its effects are not as potent as they should be. The eyes, heart, blood vessels, kidneys, and nerves may suffer due to the long-term effects of chronic hyperglycemia and the metabolic dysregulation that accompanies it. Peripheral neuropathy is the most common microvascular complication of diabetes [3,4]. Diabetic neuropathy affects many types of nerves, including sensory, motor, and autonomic. Neuropathy is common in people with diabetes, with rates ranging from 7% after a year of diagnosis to over 50% after more than 25 years [3]. One of the most significant microvascular side effects of diabetes is diabetic neuropathy, which has been associated with foot amputation, ulceration, and a poor quality of life [5,6]. Hyperglycemia causes structural and functional problems, even if the neuropathic symptoms are moderate or subclinical.

The nerve conduction study (NCS) calculates the velocities at which electrical impulses travel through the peripheral nerves. When the abnormality occurs in the axon, myelin, and nodes of Ranvier, aberrant results might be noticed [7]. In NCS, the amplitude of the nerve potential is proportional to the degree of axonal damage or loss [8]. As a result, even if the nerve damage is preclinical or pre-symptomatic, NCS can aid in the identification of peripheral nerve dysfunction [5]. Diabetes-related neuropathy is the most common cause of peripheral neuropathy [6]. In the present study, we used NCS neurophysiological parameters to investigate the influence of glycemic management on nerve physiological functioning independent of the duration of diabetes. Thus, this study was planned to assess peripheral nerve function in patients with diabetes in order to see if there exists a link between glycemic management and diabetic peripheral neuropathy.

## Materials and methods

A cross-sectional analytical research study was conducted starting from June 2021 for a period of one year in the neurophysiology lab involving 150 type 2 diabetes patients aged 35 to 60 years who were selected from the diabetic outpatient department and were divided into two groups: group A (sample size: 90 with HbA1c levels <10) and group B (sample size: 60 with HbA1c levels >10). Patients with type I diabetes, myopathy or polyneuropathy, neuromuscular transmission disorders such as myasthenia gravis, diabetic foot, a history of lumbosacral radiculopathy, or other metabolic or endocrine diseases were excluded from the research. The fasting and postprandial blood sugar, and HbA1c tests were done in all patients. Blood sugar estimation was done with the help of venous blood sampling, and a fasting level of <120 mg/dl and a post-meal level of <140 mg/dl were taken as the normal ranges of sugar [1]. HbA1c was estimated by the immunoturbidity method and an HbA1c level <6.5% was considered to be normal [9,10].

Nerve conduction studies were performed as per international standards with an RMS EMG EP Mark-II machine (RMS, Panchkula, India) and Neurosoft chrome machine The parameters that were recorded by nerve conduction studies [11] included the motor nerve conduction velocity (MNCV) of the tibial nerve, the sensory nerve conduction velocity (SNCV) of the sural nerve, tibial nerve compound muscle action potential (CMAP) amplitude, and sural nerve sensory nerve action potential amplitude (SNAP). For the tibial motor nerve conduction investigation, the recording electrode (colored black) was positioned over the abductor hallucis muscle's belly, where the CMAP was recorded. The red reference electrode was situated distally near the metatarsal head, which is an electrically neutral location. A ground electrode was positioned above the dorsum of the foot (green electrode). The distal latency, motor nerve conduction velocity (CV) of the tested segment, and CMAP amplitudes were measured following the stimulation. CV ≥41 m/s and CMAP ≥4.0 mV were taken as normal values [11] for tibial motor nerve conduction.

The measurement of the sensory nerve conduction in the sural nerve was done using antidromic surface stimulation. The reference electrode (red) was positioned 3 cm distally from the recording electrode (black), which was positioned between the lateral malleolus and the Achilles tendon. Over the Achilles tendon, a ground electrode (green) was positioned 5 cm in front of the recording electrode. The leg should be relaxed and lateral in position during the recording. The distal latency, nerve conduction velocity (SNCV) of the tested segment, and SNAP amplitudes were measured. SNCV was calculated by dividing the distance between the stimulation and recording electrodes by the peak latency. A CV of ≥40 m/s and SNAP of ≥6.0 µV were taken as normal values [11] of sural sensory nerve conduction. The outcomes are affected by improper electrode positioning, imprecise readings, and a failure to track and regulate limb temperature [12]. The sensory and motor parameters of group A (HbA1c <10) and group B (HbA1c >10) were noted and then they were compared statistically. The statistical analysis was carried out with GraphPad Prism (Dotmatics, Boston, MA) with the help of an unpaired t-test. A p-value <0.05 was considered statistically significant and a p-value <0.0001 was considered statistically very highly significant.

## Results

In this cross-sectional analytical study, 150 type II diabetic patients were included. The subjects were divided into two groups based on their HbA1c levels. Groups A and B were designated as having HbA1c levels below and above 10 respectively. There were 60 subjects in group B and 90 subjects in group A. The mean age of subjects in group A was 47.48 ± 8.14 years and that in group B was 49.23 ± 7.81 years. When the age group was compared, it was found to be similar across the two groups (p=0.1806, not significant). Of the 90 subjects in group A, there were 53 (58.8%) males and 37 (41.11%) females. Of the 60 subjects in group B, there were 35 (58.33%) males and 25 (41.66%) females (Table 1).

**Table 1:** Demographic profile of cases in group A and group B Table 1 shows the demographic profile of cases in group A (HbA1C <10) and group B (HbA1C >10). The sample size in group A is 90 and that in group B is 60. There was no significant difference between the two groups in terms of age (p=0.1806, NS) SD: standard deviation; NS: not significant

Parameter	Group A (HbA1C <10)	Group B (HbA1C >10)	P-value
Number of cases	90	60	
Age in years, mean ± SD	47.48 ± 8.14	49.23 ± 7.81	0.1806 (NS)
Sex, n (%)			
Male	53 (58.8%)	35 (58.33%)	
Female	37 (41.11%)	25 (41.66%)	

The comparison of neurophysiological parameters between the two groups was performed as shown in Table 2. MNCV in group A was 53.34 ± 8.24 m/sec as compared to 48.31 ± 9.15 m/sec in group B, which was statistically significant (p<0.05). CMAP in group A was 5.74 ± 2.88 mV and that in group B was 3.90 ± 2.71 mV, which was again statistically significant (p<0.05). In sensory nerve group neurophysiological studies on the sural nerve, it was observed that SNCV in group A was 50.19 ± 6.95 m/sec and that in group B was 41.43 ±7.07 m/sec. Also, SNAP in group A was 15.45 ±4.35 µV and that in group B was 8.33 ± 2.63 µV. Both the sensory nerve parameters showed statistically very high significance (p<0.0001).

**Table 2:** Comparison of nerve conduction study parameters between group A and group B *Statistically significant (p<0.05). **Statistically very highly significant (p<0.0001) SD: standard deviation; MNCV: motor nerve conduction velocity; CMAP: compound muscle action potential; SNCV: sensory nerve conduction velocity; SNAP: sensory nerve action potential

NCV studies	Parameter	Group A (HbA1C <10)	Group B (HbA1C >10)	P-value
	Number of cases	90	60	
Tibial motor conduction studies, mean ± SD	MNCV (m/sec)	53.34 ± 8.24	48.31 ± 9.15	0.0024*
	CMAP (mV)	5.74 ± 2.88	3.90 ± 2.71	0.0065*
Sural sensory conduction studies, mean ± SD	SNCV (m/sec)	50.19 ± 6.95	41.43 ± 7.07	<0.0001**
	SNAP (µV)	15.45 ± 4.35	8.33 ± 2.63	<0.0001**

Thus, the results of the study suggest that in motor neurophysiological parameters, there was a statistically significant difference between the findings in group A and group B. It was observed that group B had a lower CV and amplitude potential than group A, with a significant statistical difference (p<0.05). In sensory neurophysiological parameters, there was a statistically very high significant difference between the findings in group A and group B. The sensory CV and amplitude potential were lower in group B as compared to group A with a high statistically significant difference (p<0.0001). Based on these results, we ascertain that as HbA1c increased the motor and sensory CV and amplitude potential were affected significantly. It was also observed that sensory parameters (p<0.0001) were more affected than motor parameters (p<0.05).

## Discussion

The major goal of this study was to perform a cross-sectional analytical research using neurophysiological measures to examine the influence of glycemic management on nerve physiological functioning, regardless of diabetes duration. When electrophysiological values in groups A (HbA1c <10) and B (HbA1c >10) were compared (Table 2), electrophysiological parameters were found to be significantly different. The NCS studies of the subjects in group B were affected more than in group A and it was found in the analysis that for motor conduction studies, the level of significance was p<0.05 while it was p<0.0001 for sensory conduction studies. It was also observed that the sensory parameters (p<0.0001) were significantly more affected than motor parameters (p<0.05). Patients with high HbA1c levels exhibit slowing of nerve CV, which indicates myelin sheath degradation, and amplitude reduction due to axonal loss [6]. Thus, the current study shows that when HbA1c levels rise, the severity of sensory and motor axonal and demyelinating neuropathy also worsens [6]. As a result, glycemic management is linked to the development of diabetic neuropathy.

There are various studies that show that long-term diabetes and high levels of glycated hemoglobin are both linked to a high risk of neuropathy [13,14]. In univariate and multivariate regression analysis by Tkac et al., it was observed that the severity of diabetic peripheral sensorimotor polyneuropathy expressed by electrophysiologic criteria was significantly related to glycemic control in a study of patients with type 1 or type 2 diabetes [15]. Shaw et al. [16] evaluated 91 patients with neuropathy by using neuropathy disability score (NDS) and vibration perception thresholds (VPT). However, NDS was associated with age (p=0.0001) and HbA1c (pP=0.003) whereas the degree of neuropathy as evaluated by VPT score was associated with increasing glycosylated hemoglobin (p=0.02) and male sex (p=0.03). Glycated hemoglobin (GHb) was discovered to be substantially associated with fiber density in univariate and multivariate regression analysis by Perkins et al. [8] in 2001. Amthor et al. [17] found a significant reduction in nerve CV during an eight-year period in diabetic patients with a mean HbA1c of more than 10%. The Kumamoto study found that intensive insulin therapy for seven years increased type 2 diabetic patients' nerve CV and VPT compared to those receiving standard care [18]. In a research by the Diabetes Control and Complications experiment, intense insulin therapy for 6.5 years successfully reduced the incidence of neuropathy by 60% [19]. A cross-sectional, population-based study was conducted by Herman et al. [20] with the aim of describing glycaemic control and the prevalence of microvascular and neuropathic sequelae among Egyptians with confirmed diabetes, previously undiagnosed diabetes, impaired glucose tolerance, and normal glucose tolerance. Microvascular and neuropathy problems among those with documented diabetes were linked to hyperglycemia. The relationship between glycemic control and complications in type 2 diabetes mellitus was researched by Stolar [21] in 2010. He observed that HbA1C was closely associated with microvascular problems such as nephropathy, retinopathy, and neuropathy [21].

It was also observed in the present study that sensory nerve study parameters were significantly more affected as compared to motor nerve study parameters (Table 2). Our findings were similar to those of Aruna et al. who concluded that sensory nerves are more affected than motor nerves [22]. This indicates that the sensory nerves are more vulnerable to damage than motor nerves in diabetic peripheral neuropathy [22,23]. There are several other studies that found that the involvement of the sural nerve was higher as compared to other nerves [24,25]. Thus, it can be concluded that the sural nerve conduction study can evaluate the most distal segments of the extremities and can be considered an alternative method for the diagnosis of polyneuropathy in DM [22,24].

Several different pathophysiological pathways underlying diabetic neuropathy have been described. Axonal loss, endoneurial microangiopathy, and distal and sensory-predominant nerve fiber destruction are the most frequent peripheral nervous system findings in diabetes patients [26,27]. Based on this anatomic condition, Dyck et al. hypothesized that microvascular damage is the most likely source of localized fiber loss and that its aggregation appears to be the root cause of diffuse fiber loss of distal predominant axonal neuropathy in diabetes [28,29]. According to Kitano et al. [30], tissue acidosis and reduced Na/K ATPase activity are two metabolic variables that may be causing the slowing of nerve conduction. Yagihashi et al. described a number of metabolic pathways that are known to be active in chronic hyperglycemia and contribute to the onset of neuropathy [3]. Diabetes patients who have hyperglycemia experience peripheral nerve injury as a result of increased polyol pathway flux, higher formation of advanced glycation end products (AGE), increased cytokine release, activated protein kinase C, and worsened oxidative stress, among other factors [3].

Limitations

With the aid of the HbA1c levels, we examined electrodiagnostic parameters in this study based on glycemic management, regardless of the duration of diabetes. The scope of research was limited to HbA1c levels and their relationship to diabetic neuropathy in terms of glycaemic management. The fact that the study's data were cross-sectional constitutes one of the study's limitations. Therefore, long-term longitudinal research in the future may be necessary for additional assessment. The examination of individuals with severe diabetic complications may be constrained by age restrictions in the inclusion criteria (35-60 years). This could make it more difficult for us to detect any meaningful relationships between glycemic control and neurophysiological markers.

## Conclusions

The primary goal of this study was to use neurophysiological parameters irrespective of the duration of diabetes to evaluate the association between HbA1c levels and the physiological functioning of nerves. The study results revealed that as HbA1c increases the severity of neuropathy also worsens. It was also observed that sensory electrophysiological parameters were more affected than motor parameters. Thus, we conclude that glycemic control and the degree of neuropathic alterations are associated. Additionally, it implies that inadequate glycemic control should be taken into account as a risk factor for the onset of diabetic neuropathy.
